# The Associations between VEGF Gene Polymorphisms and Diabetic Retinopathy Susceptibility: A Meta-Analysis of 11 Case-Control Studies

**DOI:** 10.1155/2014/805801

**Published:** 2014-04-28

**Authors:** Liyuan Han, Lina Zhang, Wenhua Xing, Renjie Zhuo, XiaLu Lin, Yanhua Hao, Qunhong Wu, Jinshun Zhao

**Affiliations:** ^1^Department of Preventive Medicine, Zhejiang Provincial Key Laboratory of Pathophysiology, Ningbo University, Ningbo 315211, China; ^2^Department of Social Medicine, School of Public Health, Harbin Medical University, 157 Baojian Road, Harbin, Heilongjiang Province 150081, China

## Abstract

*Aims*. Published data on the associations of VEGF polymorphisms with diabetic retinopathy (DR) susceptibility are inconclusive. A systematic meta-analysis was undertaken to clarify this topic. *Methods*. Data were collected from the following electronic databases: PubMed, Embase, OVID, Web of Science, Elsevier Science Direct, Excerpta Medica Database (EMBASE), and Cochrane Library with the last report up to January 10, 2014. ORs and 95% CIs were calculated for VEGF–2578C/A (rs699947), –1154G/A (rs1570360), –460T/C (rs833061), −634G>C (rs2010963), and +936C/T (rs3025039) in at least two published studies. Meta-analysis was performed in a fixed/random effect model by using the software STATA 12.0. *Results*. A total of 11 studies fulfilling the inclusion criteria were included in this meta-analysis. A significant relationship between VEGF+936C/T (rs3025039) polymorphism and DR was found in a recessive model (OR = 3.19, 95% CI = 1.20–8.41, and *P*(*z*) = 0.01) in Asian and overall populations, while a significant association was also found between –460T/C (rs833061) polymorphism and DR risk under a recessive model (OR = 2.12, 95% CI = 1.12–4.01, and *P*(*z*) = 0.02). *Conclusions*. Our meta-analysis demonstrates that +936C/T (rs3025039) is likely to be associated with susceptibility to DR in Asian populations, and the recessive model of –460T/C (rs833061) is associated with elevated DR susceptibility.

## 1. Introduction

Type 2 diabetes (T2DM) is a metabolic disorder that has caused major public health threat throughout the world. Diabetic retinopathy (DR), one of the most prominent pathological microvascular complications of T2DM, is also the leading cause of legal blindness in working-age adults [1], but its frequency varies in different ethnicities.

Hyperglycemia has been regarded as the dominant pathogenic factor in the development and progression of DR [2]. The Wisconsin Epidemiologic Study of Diabetic Retinopathy (WESDR) showed that 28.8% of diabetic patients develop retinopathy early, whereas 22.2% with the history of diabetes irrespective of glycemic exposure do not develop retinopathy [3]. This study suggested that genetic factors could facilitate the happening of retinopathy in diabetic patients.

Currently, the pathogenesis of DR is considered to be influenced by environmental and genetic factors. Ethnic differences in the prevalence of DR may offer understandings into the relative importance of genetic or environmental risk factors. Therefore, it is important to identify molecular markers that may help in the diagnosis of DR in multiple populations.

Diabetic retinopathy is characterized by vascular permeability, increased tissue ischemia, and angiogenesis. Vascular endothelial growth factor (VEGF), a potent angiogenic and vascular permeability factor [4], plays a significant role through inducing hyperpermeability of retinal vessels, breakdown of the blood-retinal barrier, and neovascularization [5–7]; moreover, VEGF antagonists are able to reduce retinal vascular permeability and neovascularization, thus inhibiting the development of DR [8, 9]; therefore VEGF may be strongly implicated in the progression of DR.

The human VEGF gene is located on chromosome 6 (6p21.3) and highly polymorphic, especially in the promoter, the 5′-untranslated (UTR) and the 3′-untranslated region. The promoter has a single transcription start site near to a group of Sp1 binding sites and covers AP-1 and AP-2 binding sites [10], while the 3′-UTR of VEGF gene is predicted to embrace mRNA destabilizing elements which reduces VEGF mRNA under normoxic conditions and revealed to be acting in conjunction with the 5′-UTR and coding region of the gene to bring about mRNA stability during hypoxia [11].

Until now, the exact pathogenesis of DR is not yet fully clarified, despite a large number of studies on the candidate genes for the DR susceptibility in subjects of various ethnicities; however, most of the data appear to be inconclusive and require further confirmation. This may be attributed to the limited sample size and inadequate statistical power and studies with a relatively small sample size, which may have affected their reliability. Meta-analysis provides the most accurate estimate of the nature and magnitude of an effect by combining the results of multiple independent studies and has the ability to reduce the potential influence of types I and II errors that appear within individual studies [12]. Therefore, we performed a comprehensive meta-analysis to evaluate and confirm the associations of VEGF gene polymorphisms with DR susceptibility; we focused on the promoter region (−2578C/A rs699947, −1154G/A rs1570360), 5′-UTR(−634G>C rs2010963, −460T/C rs833061), and the 3′-UTR(+936C/T rs3025039) as it has been shown to be highly polymorphic and the most studied polymorphisms.

## 2. Subjects and Methods

### 2.1. Identification and Eligibility of Relevant Studies of Meta-Analysis

A systematic literature search in PubMed, Embase, OVID, Web of Science, Elsevier Science Direct, Excerpta Medica Database (EMBASE), and Cochrane Library (last search updated on January 10, 2014) was carried out to identify studies involving the associations between DR and the above mentioned VEGF polymorphisms. The language was limited to English. In this meta-analysis, the controls were patients with T2DM without DR (DWR) and the cases were T2DM patients with DR (all retinopathy, including NPDR-nonproliferative diabetic retinopathy and PDR-proliferative diabetic retinopathy). We conducted subgroup analyses stratified by status of DR (based on the studies that had defined cases solely in the presence of PDR or NPDR) [13].

The search terms were as follows: “vascular endothelial growth factor or VEGF” in integration with “polymorphism or mutation or variant” and in integration with “diabetic retinopathy or DR or NPDR or PDR” to identify all publications, which investigated the associations of the VEGF polymorphisms with DR risk in all ethnic populations. Meanwhile, we also searched the reference lists of included studies to identify other potentially eligible studies. Case reports, editorials, and reviews were excluded.

Studies included in our meta-analysis must meet the following criteria: (1) the article pertained to the above mentioned VEGF polymorphisms and DR risk; (2) sufficient data for examining odds ratios (ORs) with 95% confidence intervals (CIs); (3) genotype distributions of polymorphism of the control population were consistent with Hardy-Weinberg equilibrium (HWE).

Two investigators independently extracted data. From each study, we extracted the first author's name, year of publication, country of origin, ethnicity of samples, number of cases and controls, and the available genotype frequency of the polymorphisms. Ethnicity was classified as White or Asian. The control group sources were classified as population-based or hospital-based controls. The deviation of the genotype frequencies in the control population from HWE was calculated separately for each study.

### 2.2. Statistical Analysis

We conducted the meta-analysis using STATA software (version 12; Stata Corporation, College Station, Texas). ORs and 95% CIs were calculated to assess the strength of the associations between the VEGF polymorphisms and DR susceptibility. The pooled OR was calculated for the codominant model, dominant model, recessive model, and additive model, respectively. *P* < 0.05 was considered statistically significant.

The inconsistency index, *I* ^2^, was calculated to identify the heterogeneity [14]. The data were used with the fixed effects pooling model if there was no heterogeneity (*I* ^2^ < 50%). Alternatively, the random effects model was used (*I* ^2^ > 50%). If there is heterogeneity, the Galbraith graph was used to explore the potential sources of heterogeneity. We assessed the potential publication bias with funnel plots of the effect sizes versus the standard errors and identified the significant asymmetry by the Begg's test [15]. To test for funnel plot asymmetry, Egger's test was also performed [15]. The leave-one-out sensitivity was performed, in which the meta-analysis estimates were computed after every study being omitted in each turn [16].

## 3. Results

### 3.1. Characteristics of All Included Studies

**Figure 1:**
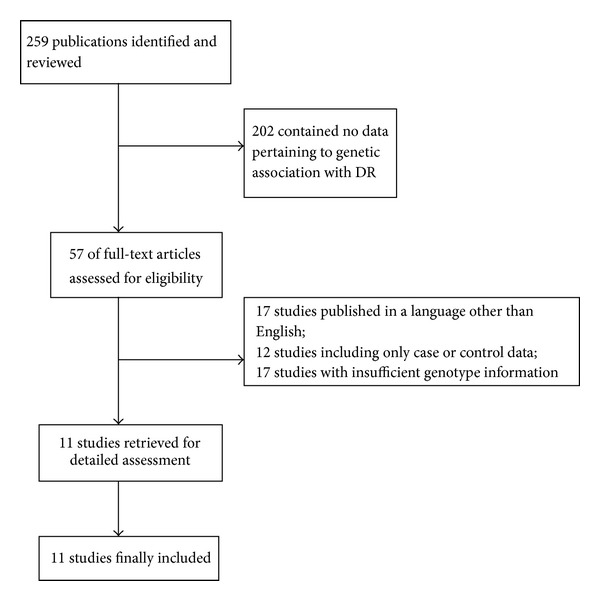
Flow chart of study selection process and included studies.

The initial search yielded 259 references. Based on titles and/or abstracts, we excluded 202 and reviewed 57 full-text reports. Applying the study inclusion criteria, 11 studies were included in this meta-analysis (Figure 1). The study selection procedure was showed in Figure 1, and the study characteristics were displayed in Tables 1 and 2.

Seven relevant studies with a total number of 1,085 cases and 1,019 controls were included in −634G>C (rs2010963) analysis [17–23]; 6 relevant studies with a total number of 887 cases and 981 controls were included in −2578C/A (rs699947) analysis [17, 20–24]; 4 relevant studies with a total number of 531 cases and 616 controls were included in +936C/T (rs3025039) analysis [19, 22, 25, 26], while 3 relevant studies with a total number of 399 cases and 347 controls were included in −460T/C (rs833061) analysis [22, 25, 27] (Table 1). The distributions of the genotypes in the control populations were consistent with HWE in all of the studies.

Of the 11 studies, 9 were hospital-based control studies [17, 18, 20–25, 27], and 2 were population-based control studies [19, 26]. A total of 8 studies included Asian individuals [17, 19, 26] and 3 included White individuals [18, 23, 24].

Three relevant studies were included in −634G>C (rs2010963) analysis (PDR versus NPDR), and 3 relevant studies were included in +936C/T (rs3025039) analysis (PDR versus NPDR) (Table 2).

### 3.2. Pooled Effects for the VEGF −634G>C (rs2010963) Polymorphism and DR Risk (DR versus DWR)

Analyses were performed for all cases with any form of DR compared with all diabetics without retinopathy (DWR). The summary results of meta-analysis for VEGF gene polymorphisms and DR risk were shown in Table 3. No significant association was detected under all genetic models in the overall populations and subgroup analysis for −634G/C (rs2010963) polymorphism (Table 3).

### 3.3. Pooled Effects for the VEGF −2578C/A (rs699947) Polymorphism and DR Risk (DR versus DWR)

Our meta-analysis did not show any significant correlations between −2578C/A (rs699947) and DR risk in hospital-based control studies, overall populations, and Asians populations, respectively (Table 3).

### 3.4. Pooled Effects for the VEGF +936C/T (rs3025039) Polymorphism and DR Risk (DR versus DWR)

Our data demonstrated that +936C/T (rs3025039) polymorphism increased the DR risk in the Asian populations (recessive model, OR = 3.19, 95% CI = 1.20–8.41, and *P*(*z*) = 0.01). The effects of the ORs and 95 CIs of the Asian populations and overall populations were the same, because all of the studies included in the +936C/T (rs3025039) were all Asian populations (Table 3).

### 3.5. Pooled Effects for the VEGF −460T/C (rs833061) Polymorphism and DR Risk (DR versus DWR)

A significant association between the −460T/C (rs833061) polymorphism and increased DR risk was detected under a recessive model (OR = 2.12, 95% CI = 1.12–4.01, and *P*(*z*) = 0.02) (Table 3).

### 3.6. Pooled Effects for the VEGF −1154G/A (rs1570360) Polymorphism and DR Risk (DR versus DWR)

There were only two studies included in the −1154G/A (rs1570360) meta-analysis; therefore the −1154G/A (rs1570360) was not further analyzed.

### 3.7. Pooled Effects for the VEGF −634G>C (rs2010963), +936C/T (rs3025039) Polymorphism and DR Risk (PDR versus NPDR)

There were only three studies included in −634G>C (rs2010963) and +936C/T (rs3025039) meta-analysis (PDR versus NPDR) (Table 2). Our meta-analysis did not show any significant correlations between VEGF −634G>C (rs2010963), +936C/T (rs3025039), and DR risk, respectively (PDR versus NPDR) (Table 4).

### 3.8. Heterogeneity Analysis

In Table 3, we have noticed that most of the comparisons had significant heterogeneity, we therefore conducted subgroup analysis with available studies, the Asian populations and hospital-based studies were mainly the sources of heterogeneity. However, in the significant comparisons, recessive model in Asian populations of the +936C/T (rs3025039) polymorphism, and recessive model of −460T/C (rs833061), heterogeneity was not found (Table 3).

### 3.9. Publication Bias

Publication bias was examined by funnel plots qualitatively and assessed by Egger's tests quantitatively. The results of Egger's regression test showed that there was no publication bias for the significant comparisons (*P* = 0.53 for the recessive model of +936C/T (rs3025039) in Asian populations; *P* = 0.66 for the recessive model of −460T/C (rs833061)) (Table 3).

Begg's test and Egger's test did not detect any significantly statistical evidence of publication bias for any of the genetic models. This indicates that the results of this meta-analysis are relatively stable and that publication bias is unlikely to have affected the results.

### 3.10. Sensitivity Analysis

We performed the sensitivity analyses by sequentially removing individual eligible study. The results indicated that the overall significance of the ORs was not altered by any single study for the recessive model of the +936C/T (rs3025039) polymorphism in Asian populations and −460T/C (rs833061) (data not shown). The sensitivity analyses also indicate that results of our study are stable and reliable.

## 4. Discussion

To our best of knowledge, this is the first meta-analysis involving the five VEGF polymorphisms at the same time. Our meta-analysis finds significant associations between +936C/T (rs3025039) and DR susceptibility in Asian populations, while Kim et al. found that +936C/T (rs3025039) polymorphism was related with DR in Korean populations [26]; however Awata et al. demonstrated nonsignificant association of this polymorphism with the progression of DR in Japanese [25]. The main reason for this discrepancy might be racial differences in the studied populations. Other reasons might be due to differences in the inclusion criteria of cases, sampling bias, sample sizes, and so forth.

The most investigated VEGF polymorphism has been −634G>C (rs2010963), with most studies showing no significant association between this polymorphism and the presence of DR; as well, the present meta-analysis confirms the nonsignificant association across all of the overall and subgroup analysis, which is consistent with the results of T. Zhao and J. Zhao meta-analysis [28]. Although we reached the same conclusion, there are some differences between us. First, eight studies were included in T. Zhao and J. Zhao meta-analysis, three of them deviated from HWE [27, 29, 30], therefore resulting in obvious heterogeneity. Second, an expanding body of literature on this topic has been published since 2010, but unfortunately was not included in the T. Zhao and J. Zhao meta-analysis. Besides, T. Zhao and J. Zhao also included the study conducted by Kangas-Kontio et al. [31], which was inappropriate, because the diabetic patients also included type 1 diabetes in that study. We believe our results are more reliable and stable based on the sample size, thoughtful design, and strict criterion for the included studies.

Contrary to our meta-analysis, Qiu et al. conducted a similar meta-analysis only on −634G>C polymorphism and DR, which involved a total of 1525 DR cases and 1422 DWR controls in 9 independent studies, they observed a significant relationship between −634G>C (rs2010963) polymorphism and DR in an allelic genetic model (OR: 1.13) and a recessive genetic model (OR: 1.26). However, the genotypes deviation of the controls in Yang et al.'s study was not consistent with HWE [32] but was still included in Qiu et al. meta-analysis [33], though they stated that all the studies included were not deviated from HWE. Moreover, the study performed by Errera et al. was inappropriate to include in their meta-analysis, because the T2DM patients were divided into patients with PDR and patients without PDR in that study [34]; as we all know, DR consists of PDR and NPDR, the cases and controls were DR and DWR in Qiu et al. meta-analysis respectively, and a significant association was evidenced in Errera et al. study (with the second largest weight from the forest plot) in Qiu et al. meta-analysis; therefore the results of Qiu et al. meta-analysis maybe biased and unreliable.

Some possible limitations of our meta-analysis should be taken into consideration. First, the conclusion was based on a relatively small number of participants. Second, potential publication biases may exist in this meta-analysis because studies excluded the non-English-language publications. Third, this meta-analysis was based on unadjusted data due to a lack of detailed genotype information stratified by many variables (gender, age, etc.) in original articles, and a more precise analysis would have been performed if all individual raw data had been available.

In spite of these potential limitations, our meta-analysis has some strength. First, we sought to find as many publications as we could by searching various databases. The sufficient number of cases and controls were pooled from multiple studies, which apparently increased the statistical power of our analysis. Second, in order to minimize the potential bias, we designed a rigorous protocol and utilized explicit methods for the literature search, study selection, data extraction, and statistical analysis. The symmetry of the funnel plot suggests that bias is less likely to have appeared, indicating that the pooled results of our analysis may be unbiased. Third, no publication bias was detected among the pooled results. Last, we considered not only association between the most investigated −634G>C (rs2010963) and DR susceptibility but also paid attention to the impact of other VEGF polymorphisms −2578C/A (rs699947), −1154G/A (rs1570360), −460T/C (rs833061), and +936C/T (rs3025039). We could therefore give a more complete picture on the role of VEGF polymorphisms contributing to DR risk.

In conclusion, our meta-analysis revealed some significant associations between VEGF polymorphisms and DR susceptibility. However due to the relatively small sample size in this meta-analysis, in order to reach a more definitive conclusion, further studies based on larger sample size and substantiation of the variations through functional studies are still needed.

## Figures and Tables

**Table 1:** Characteristics of all studies assessing the relationship between VEGF and diabetic retinopathy (DR versus DWR).

First author	Year	Country/racial decent	Study design	Cases^a^ (DR)	Controls^a^ (DWR)		Cases			Controls		HWE
						11^b^	12^b^	22^b^	11^b^	12^b^	22^b^	*X* ^2^ control population
−634 G>C (rs2010963)												
Awata [17]	2005	Japan (Asian)	Hospital based controls	175	203	46	91	38	75	95	30	0.99
Globočnik Petrovič [18]	2008	Slovene (Caucasian)	Hospital based controls	206	143	79	103	24	61	67	15	0.58
Uthra [19]	2008	India (Asian)	Population based controls	131	82	60	51	9	44	29	6	0.69
Nakamura [20]	2009	Japan (Asian)	Hospital based controls	177	292	63	79	34	84	146	59	0.75
Chun [21]	2010	Korea (Asian)	Hospital based controls	253	134	85	125	43	43	69	22	0.51
Yang [22]	2011	China (Asian)	Hospital based controls	129	139	36	74	19	39	72	26	0.47
Bleda [23]	2012	Spain (Caucasian)	Hospital based controls	14	26	7	5	2	10	11	5	0.53

–2578 C/A (rs699947)												
Awata [17]	2005	Japan (Asian)	Hospital based controls	175	203	95	70	10	93	91	19	0.62
Abhary [24]	2009	Australia (Caucasian)	Hospital based controls	139	187	31	74	31	45	91	45	0.94
Nakamura [20]	2009	Japan (Asian)	Hospital based controls	177	292	85	70	22	163	107	22	0.44
Chun [21]	2010	Korea (Asian)	Hospital based controls	253	134	123	115	15	92	36	6	0.31
Yang [22]	2011	China (Asian)	Hospital based controls	129	139	66	47	16	82	51	5	0.38
Bleda [23]	2012	Spain (Caucasian)	Hospital based controls	14	26	0	12	2	8	10	8	0.23

+936 C/T (rs3025039)												
Awata [25]	2002	Japan (Asian)	Hospital based controls	150	118	93	47	10	85	31	2	0.66
Uthra [19]	2008	India (Asian)	Population based controls	131	82	111	19	0	65	17	0	0.29
Kim [26]	2009	Korea (Asian)	Population based controls	121	277	55	63	3	226	51	0	0.09
Yang [22]	2011	China (Asian)	Hospital based controls	129	139	83	42	3	89	47	3	0.25

−460 T/C (rs833061)												
Awata [25]	2002	Japan (Asian)	Hospital based controls	150	118	79	58	13	52	57	9	0.21
Suganthalakshmi [27]	2006	India (Asian)	Hospital based controls	120	90	36	81	3	61	29	0	0.06
Yang [22]	2011	China (Asian)	Hospital based controls	129	139	65	46	16	81	52	5	0.33

DR: diabetic retinopathy; DWR: diabetic without retinopathy; HWE: Hardy-Weinberg equilibrium.

^a^Sample size.

^b^11: wild-type homozygote; 12: heterozygote; 22: variant homozygote.

**Table 2:** Characteristics of all studies assessing the relationship between VEGF and diabetic retinopathy (PDR versus NPDR).

First author	Year	Country/racial decent	Study design	Cases^a^ (PDR)	Controls^a^ (NPDR)		Cases			Controls	
						11^b^	12^b^	22^b^	11^b^	12^b^	22^b^
−634 G>C (rs2010963)											
Awata [25]	2002	Japan (Asian)	Hospital based controls	70	80	24	30	16	14	51	15
Uthra [19]	2008	India (Asian)	Population based controls	44	87	21	17	3	39	33	7
Chun [21]	2010	Korea (Asian)	Hospital based controls	145	108	48	73	24	37	52	19

+936 C/T (rs3025039)											
Awata [25]	2002	Japan (Asian)	Hospital based controls	70	80	44	20	6	49	27	4
Uthra [19]	2008	India (Asian)	Population based controls	44	87	35	9	0	76	10	0
Kim [26]	2009	Korea (Asian)	Population based controls	37	84	15	19	3	40	44	15

PDR: proliferative diabetic retinopathy; NPDR: nonproliferative diabetic retinopathy.

^a^Sample size.

^b^11: wild-type homozygote; 12: heterozygote; 22: variant homozygote.

**Table 3:** Meta-analysis of the associations between the VEGF polymorphisms and DR risk (DR versus DWR).

Variables	*n* ^a^	Variant homozygote versus wild-type homozygote				Heterozygote versus wild-type homozygote				Dominant model				Recessive model				Additive model			
		OR (95% CI)	*P*(*z*)	*I* ^2^ (%)	Egger's test *P*	OR (95% CI)	*P*(*z*)	*I* ^2^ (%)	Egger's test *P*	OR (95% CI)	*P*(*z*)	*I* ^2^ (%)	Egger's test *P*	OR (95% CI)	*P*(*z*)	*I* ^2^ (%)	Egger's test *P*	OR (95% CI)	*P*(*z*)	*I* ^2^ (%)	Egger's test *P*
−634 G>C (rs2010963)																					
Overall	7	1.07 (0.81–1.40)	0.61	17.8	0.72	1.06 (0.87–1.29)	0.54	17.6	0.88	1.04 (0.86–1.24)	0.67	30.9	0.85	1.05 (0.82–1.33)	0.69	0	0.55	1.04 (0.92–1.19)	0.47	28.5	0.80
Asian	5	1.06 (0.71–1.58)	0.75	40.6	0.97	1.04 (0.84–1.30)	0.68	39.2	0.40	1.07 (0.79–1.44)	0.66	50.6	0.53	1.04 (0.80–1.36)	0.71	0	0.75	1.04 (0.85–1.27)	0.66	48.3	0.78
Hospital based	6	1.07 (0.81–1.41)	0.63	31.5	0.71	1.03 (0.84–1.27)	0.72	26.7	0.79	1.04 (0.86–1.27)	0.63	42.2	0.74	1.05 (0.82–1.35)	0.67	0	0.58	1.03 (0.91–1.18)	0.57	39.1	0.71

–2578 C/A (rs699947)																					
Overall	6	1.47 (0.81–2.67)	0.20	59.6	0.47	1.31 (0.87–1.96)	0.19	70.0	0.34	1.33 (0.89–1.99)	0.15	72.1	0.44	1.17 (0.68–1.99)	0.56	58.9	0.98	1.24 (0.92–1.66)	0.14	72.2	0.62
Asian	4	1.59 (0.71–3.56)	0.25	71.7	0.77	1.26 (0.79–2.01)	0.32	77.3	0.73	1.32 (0.82–2.13))	0.24	80.3	0.69	1.45 (0.71–2.95)	0.29	65.1	0.80	1.29 (0.87–1.92)	0.19	81.5	0.54
Hospital based	6	1.47 (0.81–2.67)	0.20	59.6	0.47	1.31 (0.87–1.96)	0.19	70	0.34	1.33 (0.89–1.99)	0.15	72.1	0.44	1.17 (0.68–1.99)	0.56	58.9	0.98	1.24 (0.92–1.66)	0.14	72.2	0.62

+936 C/T (rs3025039)																					
Overall	4	3.73 (0.76–18.25)	0.10	50.7	0.48	1.47 (0.59–3.66)	0.40	90.8	0.29	1.54 (0.62–3.85)	0.34	91.2	0.33	3.19 (1.20–8.41)	0.01*	32.7	0.53	1.49 (0.71–3.12)	0.28	89.7	0.34
Asian	4	3.73 (0.76–18.25)	0.10	50.7	0.48	1.47 (0.59–3.66)	0.40	90.8	0.29	1.54 (0.62–3.85)	0.34	91.2	0.33	3.19 (1.20–8.41)	0.01*	32.7	0.53	1.49 (0.71–3.12)	0.28	89.7	0.34

–460 T/C (rs833061)																					
Overall	3	2.46 (0.66–9.11)	0.17	64	0.56	1.50 (0.50–4.51)	0.46	92.1	0.11	1.57 (0.51–4.89)	0.42	93	0.27	2.12 (1.12–4.01)	0.02*	42.4	0.66	1.53 (0.76–3.07)	0.22	88.3	0.25

^a^Number of studies.

*P*(*z*): *z* test used to determine the significance of the overall OR.

*I* ^2^: inconsistency index; random-effects model was used when *I* ^2^ test >50%; otherwise, fix-effects model was used.

NA: Not available.

*Significant results.

**Table 4:** Meta-analysis of the associations between the VEGF polymorphisms and DR risk (PDR versus NPDR).

Variables	*n* ^a^	Variant homozygote versus wild-type homozygote				Heterozygote versus wild-type homozygote				Dominant model				Recessive model				Additive model			
		OR (95% CI)	*P*(*z*)	*I* ^2^ (%)	Egger's test *P*	OR (95% CI)	*P*(*z*)	*I* ^2^ (%)	Egger's test *P*	OR (95% CI)	*P*(*z*)	*I* ^2^ (%)	Egger's test *P*	OR (95% CI)	*P*(*z*)	*I* ^2^ (%)	Egger's test *P*	OR (95% CI)	*P*(*z*)	*I* ^2^ (%)	Egger's test *P*
−634 G>C (rs2010963)																					
Overall	3	0.62 (0.36–1.06)	0.08	0	0.63	0.73 (0.36–1.46)	0.37	64.5	0.52	0.76 (0.43–1.34)	0.35	52.9	0.51	1.02 (0.63–1.65)	0.90	0	0.84	0.91 (0.70–1.17)	0.47	0	0.70

+936 C/T (rs3025039)																					
Overall	3	0.93 (0.37– 2.33)	0.89	27.1	NA	1.11 (0.70– 1.77)	0.64	0	0.05	1.1 (0.70– 1.72)	0.66	0	0.19	0.89 (0.37–2.13)	0.80	46.3	NA	1.04 (0.73–1.5)	0.80	0	0.29

^a^Number of studies.

*P*(*z*): *z* test used to determine the significance of the overall OR.

*I* ^2^: inconsistency index; random-effects model was used when *I* ^2^ test <50%; otherwise, fix-effects model was used.

NA: not available.
